# Prediction of local recurrence risk after neoadjuvant chemotherapy in patients with primary breast cancer: Clinical utility of the MD Anderson Prognostic Index

**DOI:** 10.1371/journal.pone.0211337

**Published:** 2019-01-31

**Authors:** Laura L. Michel, Laura Sommer, Rosa González Silos, Justo Lorenzo Bermejo, Alexandra von Au, Julia Seitz, André Hennigs, Katharina Smetanay, Michael Golatta, Jörg Heil, Florian Schütz, Christof Sohn, Andreas Schneeweiss, Frederik Marmé

**Affiliations:** 1 National Center for Tumor Diseases, University Hospital Heidelberg, Heidelberg, Germany; 2 Department of Obstetrics and Gynecology, University Hospital Heidelberg, Heidelberg, Germany; 3 Institute of Medical Biometry and Informatics, University of Heidelberg, Heidelberg, Germany; University of North Carolina at Chapel Hill School of Medicine, UNITED STATES

## Abstract

**Background:**

Locoregional recurrence after neoadjuvant chemotherapy for primary breast cancer is associated with poor prognosis. It is essential to identify patients at high risk of locoregional recurrence who may benefit from extended local therapy. Here, we examined the prediction accuracy and clinical applicability of the MD Anderson Prognostic Index (MDAPI).

**Methods:**

Prospective clinical data from 456 patients treated between 2003 and 2011 was analyzed. The Kaplan-Meier method was used to examine the probabilities of locoregional recurrence, local recurrence and distant metastases according to individual prognosis score, stratified by type of surgery (breast conserving therapy or mastectomy). The possible confounding of the relationship between recurrence risk and MDAPI by established risk factors was accounted for in multiple survival regression models. To define the clinical utility of the MDAPI we analyzed its performance to predict locoregional recurrence censoring patients with prior or simultaneous distant metastases.

**Results:**

Mastectomized patients (42% of the patients) presented with more advanced tumor stage, lower tumor grade, hormone-receptor positive disease and consequently lower pathological complete response rates. Only a few patients presented with high-risk scores (2,7% MDAPI≥3). All patients with high-risk MDAPI score (MDAPI ≥3) who developed locoregional recurrence were simultaneously affected by distant metastases.

**Conclusion:**

Our data do not support a clinical utility of the MDAPI to guide local therapy.

## Introduction

Locoregional recurrence (LRR) is associated with poor overall survival (OS).[1,2] It is essential to identify patients at high risk of LRR that might benefit from more radical local treatment (*e*.*g*. mastectomy or extended radiation fields) but at the same time to avoid overtreatment.

Neoadjuvant chemotherapy (NAC) is the treatment of choice for locally advanced primary breast cancer (PBC) but is also frequently used for early disease.[3,4] NAC increases breast conservation rates and offers the unique opportunity to evaluate response to treatment *in vivo*.

Chen et al. developed the MD Anderson Prognostic index (MDAPI) to identify patients that may benefit from an extended local therapy based on four independent risk factors for LRR after NAC and breast conserving therapy (BCT).[5] The MDAPI is composed of clinical nodal status, residual pathologic tumor size, pattern of residual disease and lymphovascular space invasion in the surgical specimen. Patients are assigned to a score between 0 and 4.[5]

However, only few reports have validated the MDAPI with conflicting results.[6–8]

Huang et al., assessed the MDAPI in 815 patients that received NAC followed by BCT or mastectomy (ME). For the few patients in the high-risk group (MDAPI 3/4), LRR rates were significantly lower for mastectomized patients, suggesting that the score might help to select patients for ME.[6]

Akay et. al validated the prognostic value of the MDAPI with regards to LRR in an independent cohort of 551 patients treated with BCT or ME. Among patients with high MDAPI (MDAPI 3/4), those treated with BCT (n = 13) had a significantly higher risk of LRR compared to those receiving ME (n = 59) (5-year LRR: 32%; *vs*. 6%).[8]

In contrast, Ishitobi et al. could not confirm a prognostic value of MDAPI to predict ipsilateral breast tumor recurrence (IBTR) in 375 patients who underwent BCT after NAC.

In all of these studies LRRs were counted as events regardless of whether they were the first sites of recurrence or occurred concomitantly with or after distant metastases (DM). However, preventing LRRs in patients with simultaneous or pre-existing DM is less important. In our study, we investigate the ability of the MDAPI score to predict the risk of LRR focusing on LRR as the first site of recurrence.

## Materials and methods

### Study cohort

Patient and tumor characteristics, therapy and follow-up of all patients referred to the Heidelberg breast cancer unit for diagnosis and treatment of PBC have been prospectively documented since January 1^st^, 2003 in our database. The study was approved by the ethics committee of the medical faculty, University of Heidelberg and was conducted according to the principles of the Declaration of Helsinki. Written informed consent was obtained from all participants.

We identified all patients treated with NAC between 2003 and 2011. To ensure a follow-up time of at least 5 years, patients diagnosed after 2011 were not enrolled in the study. Patients with DM, non-invasive, bilateral, recurrent or inflammatory disease or incomplete therapy (R1 resection, incomplete axillary staging, less than 50% of the planned chemotherapy, refusal of radiotherapy) were excluded.

### Diagnostic and therapeutic procedures

Patients were assessed by physical examination, ultrasound of the breast and axillary nodes and mammography. Histology was obtained by core needle biopsy prior to NAC. Tumors were classified according to the St. Gallen surrogate definition for intrinsic subtypes. [9,10] DM were excluded by imaging. The 6^th^ edition of the American Joint Committee on Cancer Breast Cancer Staging System was used for clinical and pathological staging. All (neo)adjuvant and locoregional therapies were determined by an interdisciplinary tumor board.

Chemotherapy regimens followed our institutional protocols and were anthracycline- and/or taxane-based. 12,4% of HER2 positive patients were treated before the approval of trastuzumab in 2005/2006 and did not receive trastuzumab. Surgery was performed within four weeks after chemotherapy. Patients with clinically node-negative disease underwent sentinel lymph node biopsy before NAC. Patients with involved sentinel nodes or clinically node-positive disease received complete axillary dissection at the time of breast surgery.

Pathological complete response (pCR) was defined as no residual invasive cancer in the breast and lymph nodes. Adjuvant radiotherapy and endocrine therapy was performed according to national guidelines.

### Statistical methods

The MDAPI was calculated for each patient as previously described.[5] Patients were grouped into 3 risk groups; low-risk: MDAPI 0&1, intermediate-risk: 2, and high-risk: 3&4, analogous to previous publications.[5,7,8] A formal sample size calculation was not performed, as the distribution to the specific risk groups was a priori unknown and we wanted to include all patients with a long enough follow-up making up a modern and large enough cohort to allow to explore the practical utility of the score.

We performed uni- and multivariate analysis of the entire cohort and stratified by type of surgery (BCT/ME) to validate the prognostic performance of the MDAPI and to identify additional risk factors for LRR, defined as ipsilateral recurrence within the breast, the thoracic wall or the axillary lymph nodes, LR, defined as recurrence within the ipsilateral breast or thoracic wall and DM. To test the clinical utility of MDAPI, LRR estimates were calculated 1.) counting LRR as an event regardless if it was the only or first site of recurrence, and 2.) censoring patients at the time of diagnosis of DM if these occurred prior, at the same time or within up to 3 months after LRR.

Patient and tumor characteristics in women undergoing BCT *vs*. ME were compared using chi-square tests. Time to LR, LRR or DM was calculated from the start of therapy. Survival times of patients who were alive and did not experience specific (locoregional, local or distant disease) recurrences at last follow up were considered censored.

Kaplan-Meier curves, log-rank tests and univariate survival regression models were used to investigate the association between the risk of recurrence and MDAPI, for all patients and stratified by type of surgery. Multiple survival regression analyses were conducted to adjust for the possible confounder effects of established prognostic factors. All statistical tests were two-sided and probability values smaller than 0.05 were considered statistically significant. Statistical analyses were conducted using SAS version 9.4 and survival curves were drawn using the R software environment for statistical computing.

## Results

**Fig 1:**
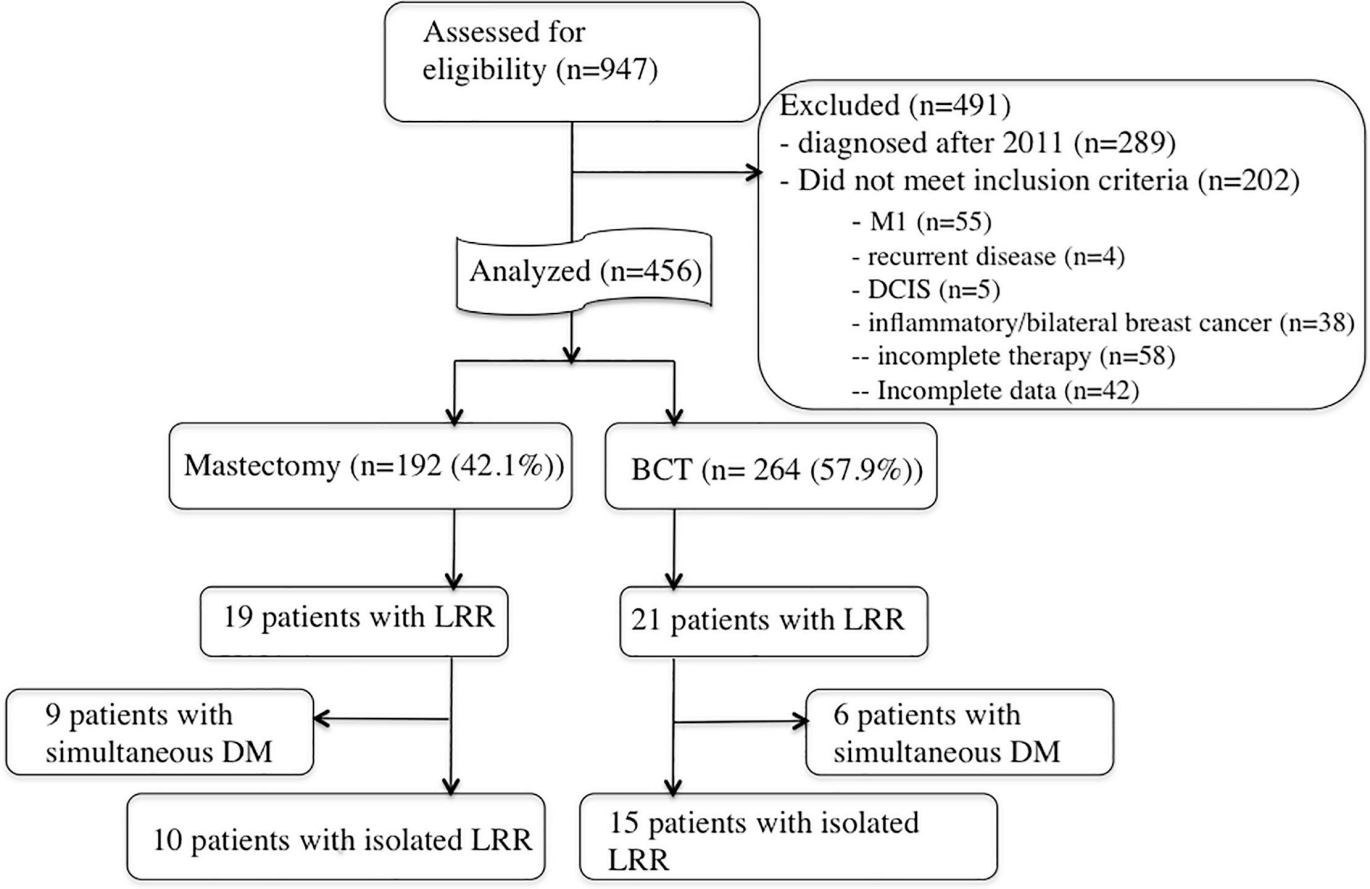
CONSORT diagram.

456 patients matched all in- and exclusion criteria (Fig 1). Patient and tumor characteristics are summarized in Table 1. Median follow up was 59 months (range, 6–142 months). 264 patients (57.9%) were treated with BCT and 192 (42.1%) with ME.

Patients treated with ME had more advanced disease (cT3/4: 43% *vs*. 14%; cN+: 28% *vs*. 11%; clinical AJCC stage III: 41% *vs*. 14%; pathological AJCC stage III: 30% *vs*. 8%), lower tumor grades (G3: 45% *vs*. 61%), more often HR-positive tumors (60% *vs*. 44%) and consequently lower pCR rates (16% *vs*. 26%) than patients treated with BCT.

**Table 1:** Main patient and tumor characteristics in the investigated collective and stratified by surgical procedure.

Variable	Level	Patients	BET	%	ME	%	Pvalue
**Age**	-41	124	60	22.7	64	33.3	0.09
	42–47	103	64	24.2	39	20.3	
	48–56	118	70	26.5	48	25.0	
	57+	111	70	26.5	41	21.4	
**cT**	0–1	31	19	7.2	12	6.3	**<0.0001**
	2	305	208	78.8	97	50.5	
	3	93	31	11.7	62	32.3	
	4	27	6	2.3	21	10.9	
**cN**	0	190	130	49.2	60	31.3	**0.001**
	1	231	119	45.1	112	58.3	
	2	14	7	2.7	7	3.7	
	3	21	8	3.0	13	6.8	
**AJCC-CS**	IA	15	8	3.0	7	3.7	**<0.0001**
	IIA	156	112	42.4	44	22.9	
	IIB	170	107	40.5	63	32.8	
	IIIA	72	23	8.7	49	25.5	
	IIIB	22	6	2.3	16	8.3	
	IIIC	21	8	3.0	13	6.8	
**Mol. ST**	Her2	50	27	10.9	23	12.8	0.09
	LmA	67	36	14.5	31	17.2	
	LmBneg	123	64	25.8	59	32.8	
	LmBpos	67	38	15.3	29	16.1	
	TNBC	121	83	33.5	38	21.1	
**Grading**	1	8	6	2.3	2	1.1	**0.001**
	2	197	95	36.7	102	54.3	
	3	242	158	61.0	84	44.7	
**ER**	0	181	117	44.8	64	33.5	**0.02**
	1	271	144	55.2	127	66.5	
**PR**	0	223	147	56.3	76	39.8	**0.001**
	1	229	114	43.7	115	60.2	
**Her2**	0	346	202	77.7	144	75.4	0.60
	1	105	58	22.3	47	24.6	
**Ki67%**	0–25	156	86	38.6	70	42.4	0.30
	25–50	106	56	25.1	50	30.3	
	50–75	57	35	15.7	22	13.3	
	75–100	69	46	20.6	23	13.9	
**ypT**	0	118	86	33.1	32	16.8	**<0.0001**
	1	179	112	43.1	67	35.1	
	2	95	47	18.1	48	25.1	
	3	33	2	0.8	31	16.2	
	4	4	1	0.4	3	1.6	
	is	22	12	4.6	10	5.2	
**ypN**	0	244	160	70.8	84	47.2	**<0.0001**
	1	105	57	25.2	48	27.0	
	2	39	6	2.7	33	18.5	
	3	16	3	1.3	13	7.3	
**AJCC-PS**	0	130	91	34.7	39	20.6	**<0.0001**
	IA	117	79	30.2	38	20.1	
	IB	10	6	2.3	4	2.1	
	IIA	90	61	23.3	29	15.3	
	IIB	35	13	5,0	22	11.6	
	IIIA	50	8	3.0	42	22.2	
	IIIB	3	1	0.4	2	1.1	
	IIIC	16	3	1.2	13	6.9	
**pCR**	0	356	195	73.9	161	83.9	**0.01**
	1	100	69	26.1	31	16.2	
**L-Status**	0	374	235	89.0	139	72.4	**<0.0001**
	1	82	29	11.0	53	27.6	
**Multifocal**	0	382	236	89.4	146	76.0	**<0.0001**
	1	74	28	10.6	46	24.0	
**MDAPI**	0	225	166	62.9	59	30.7	**<0.0001**
	1	154	77	29.2	77	40.1	
	2	65	18	6.8	47	24.5	
	3	9	3	1.1	6	3.1	
	4	3	0	0.0	3	1.6	

Abbreviations: AJCC-CS: American Joint Committee on Cancer (AJCC)–clinical stage; Mol.ST: molecular subtype, ER: estrogen receptor; PR: progesterone receptor, AJCC-PS: AJCC—pathological stage; Bold represents probability values under 0.05

21.9% of patients achieved a pCR (ypT0/is ypN0). pCR rates were highest in luminal B/Her2 positive (45.1%) and HER2 positive/non-luminal tumors (40,7%) and lowest in luminal A-like tumors (4.3%).

All patients with BCT were treated with adjuvant radiotherapy to the breast with tangential fields. 82.3% of ME patients received radiotherapy to the chest wall and 49% of all patients received radiotherapy to the regional lymph nodes, including all patients with an MDAPI 4, 88% with MDAPI 3, 71% with MDAPI 2, 66% with MDAPI 1 and 34.5% with MDAPI 0.

Most patients had low (379, 83%) and intermediate-risk (65, 14%) MDAPI scores. Patients with BCT had lower MDAPI compared to mastectomized patients (low: 92% *vs*. 71%; intermediate: 6.8% *vs*. 24.5% and high-risk: 1.1% vs. 4.7%; p<0.0001). Only 1.1% of patients with BCT were classified as MDAPI high-risk. 75% of MDAPI high-risk and 72% of MDAPI intermediate-risk patients received ME, compared to only 35% of MDAPI low-risk patients. Numbers in the MDAPI high-risk group were overall small (12 patients, 2.6%), limiting statistical power to identify potential treatment effects in this subgroup.

Within the follow up time 27 (5.9%) and 40 (8.7%) patients developed LRs and LRRs, respectively, whereas 94 (20.6%) presented with DM. 15 (37.5%) LRRs were diagnosed after or simultaneously (within 3 months) with DM. 25 (5.5%) patients presented with isolated LRR (Fig 1).

**Fig 2:**
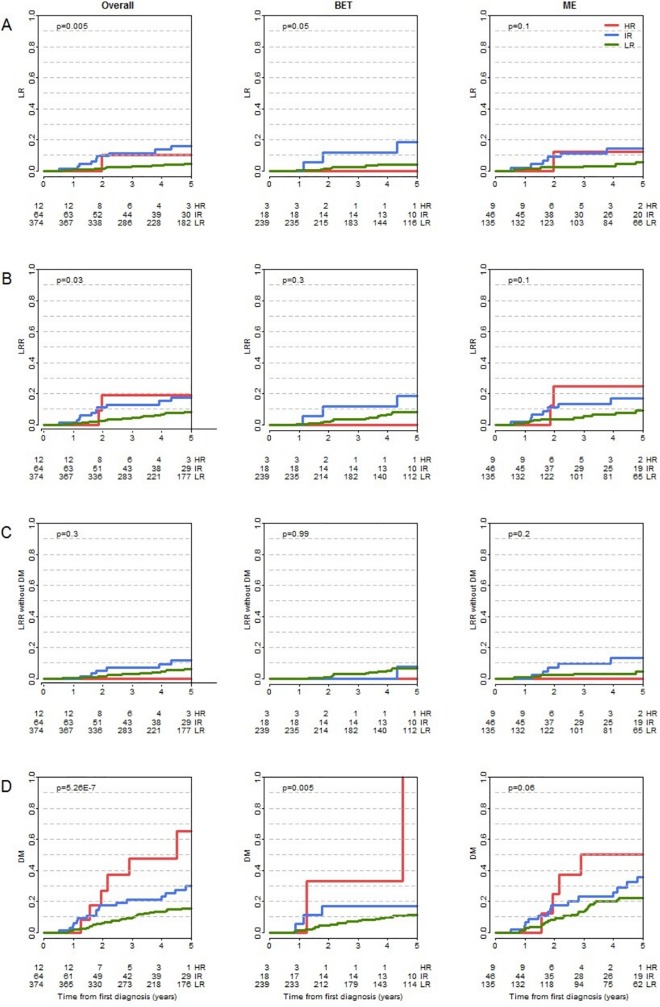
Kaplan-Meier curves for LR (A), LRR (B), LRRw/oDM (C) and DM (D) stratified by MDAPI score groups and surgical procedure (BCT/ME).

To evaluate the local risk of recurrence we calculated 5-year LR-free survival (LRFS) rates and 5-year LRR-free survival (LRRFS) rates stratified by surgical procedure. The overall 5-year LRFS rate was 94% (95% CI: 91% to 96%). The corresponding rates stratified by MDAPI risk groups were 95%, 84%, and 89% for the low, intermediate and high-risk group, respectively (p = 0.005; Fig 2A).

Differences in 5-year LRFS rates between BCT (95%) and ME (92%) did not reach statistical significance. Also, patient stratification according to MDAPI revealed no differences between BCT and ME in neither risk group (low: 96% *vs*. 95%; intermediate: 83% *vs*. 84%; high-risk: 100% *vs*. 85%).

None of the three patients with a high-risk MDAPI treated by BCT had LR whereas 1 out of 9 (15%) mastectomized patients with high-risk MDAPI had a LR. However, numbers are too small to draw conclusions with regard to potential therapy effects in the high-risk MDAPI group (S1 Table).

Kaplan-Meier curves for LRR irrespective of previous or synchronous DM are shown in Fig 2B.

The Kaplan-Meier 5-year LRRFS estimate was 92%, 81% and 77% for the low, intermediate and high-risk group, respectively (p = 0.03). Within the low and intermediate-risk groups no significant differences were found in 5-year LRRFS estimates between the BCT and ME groups (92% *vs*. 91%, 81% *vs*. 82%). Numbers in the high-risk group were too small to allow formal statistical analysis, however none of the three BCT patients in this group had LRR compared to 2 out of 9 patients with ME (S1B Table).

To evaluate the risk of DM we calculated 5-year DMFS rates (Fig 2D). The MDAPI correlated significantly with the DMFS in the overall group (low: 86%, intermediate: 66%, high-risk: 39%; p = <0.001). Patients with BCT had higher rates of DMFS compared to mastectomized patients in the overall group (87% *vs*. 73%) as well as in the low and intermediate-risk MDAPI groups, but not in the high-risk group (low: 89% *vs*. 79%; intermediate: 77% *vs*. 62%; high-risk: 28% *vs*. 42%) (S1 Table).

To focus on isolated LRR events, the event of LRR was only counted if no DM were diagnosed before, simultaneously or within 3 months after diagnosis of LRR (LRRw/oDM), (Fig 2C). 25 patients presented with LRRw/oDM. The overall 5-year LRRw/oDM-free survival rate was 93%. LRRw/oDM for patients with low and intermediate MDAPI differed numerically but results did not reach statistical significance: overall: 94% *vs*. 89% (p = 0.3), BCT: 93% *vs*. 94% (p = 0.99), ME: 96% *vs*. 87% (p = 0.2). No event occurred in the high-risk group (12 patients), indicating that all LRR events in the high-risk group described in the analyses before occurred in patients with prior or simultaneous DM (S1C Table).

Univariate survival analyses revealed that ER status, lymph node status and multifocal lesions were associated with LRR-risk. The MDAPI showed a poor ability to discriminate patients at high risk of LRR (p = 0.07) (Table 2).

**Table 2:** Results from univariate and multiple survival analyses on LRR.

				Univariate				Multiple			
Variable	Level	Patients	Events	HR	95%	CI	Pvalue	HR	95%	CI	Pvalue
Age	-41	123	18	Ref.			0.09	Ref.			0.10
	42–47	103	9	0.61	0.27	1.38		0.62	0.27	1.44	
	48–56	116	8	0.51	0.22	1.18		0.60	0.25	1.40	
	57+	108	5	0.31	0.11	0.83		0.27	0.09	0.82	
ER	0	177	20	Ref.			**0.04**				
	1	269	19	0.52	0.27	0.97					
L-Status	0	369	26	Ref.			**0.005**				
	1	81	14	2.56	1.34	4.92					
Multifocal	0	376	29	Ref.			**0.03**	Ref.			**0.01**
	1	74	11	2.14	1.06	4.29		2.50	1.20	5.20	
MDAPI	0	220	14	Ref.			0.07				
	1	154	13	1.30	0.61	2.77					
	2	64	11	2.71	1.23	5.99					
	3	9	2	4.42	1.00	19.5					
	4	3	0								

Abbreviations: HR: Hazard ratio; CI: confidence interval; ER: estrogen receptor

Bold represents probability values under 0.05

Survival differences among subtypes did not reach statistical significance. This is mostly due to the limited sample sizes of separated MDAPI high risk groups. Therefore, the molecular subtypes were not considered in the multiple survival analyses (Table 2).

## Discussion

In the present study we aimed to investigate the clinical utility of the MDAPI to guide local therapy based on a patient cohort as up-to-date and as large as possible. To ensure a patient follow-up of at least 5 years, we excluded all patients diagnosed after 2011.

Chen et al. developed the MDAPI to stratify patients undergoing BCT with respect to their risk of local recurrence. This work demonstrated that patients with high MDAPI scores are at higher risk of local recurrence [5,11]. The question that arises from this observation inevitably is, whether such patients would benefit from more radical local therapies, e.g. mastectomy. In fact two subsequent studies on the MDAPI also included mastectomized patients following its initial development and confirmed this hypothesis[6,8]. Both studies showed that in patients with an MDAPI of 3–4, mastectomy was associated with significantly lower rates of local recurrence.

We wanted to validate the clinical utility and potential impact on local management of the MDAPI in a modern world clinical setting and therefore also included mastectomized patients. By comparing local recurrence rates of BCT and mastectomized patients within the same MDAPI group we intended to investigate whether specific MDAPI groups benefit from extended surgical therapy.

We found that patients treated with ME had locally more advanced tumors, a lower tumor grade, more often PR-positive tumors and were less likely to achieve a pCR. Patients with lower tumor grades and HR-positive disease have worse tumor response to NAC which might explain the observed higher ME rates in these patients.[4] ME was associated with higher MDAPI scores. The MDAPI was not used for clinical decision making in our study, however, all 4 variables constituting the score were significantly associated with ME, indicating, that these factors play an important role in clinical decision making for locoregional therapy in our clinical practice.

The MDAPI did not discriminate patients at high risk of LR in a useful manner: High-risk patients had even slightly higher 5-year LRFS rates (89%) compared to the intermediate-risk group (84%). Similar results were observed in the ME group. The high-risk-MDAPI group of BCT patients was comprised of only three patients (out of an entire 450) and none of these experienced either an LR or LRR event (S1 Table).

We found no significant differences in the LRRFS between BCT and ME patients in the low and intermediate-risk group. Due to the low number of patients (n = 12) and events (n = 3) in the high-risk group we were not able to draw conclusions for this group, even though all local and locoregional events occurred in mastectomized patients and all of these also experienced prior or synchronous DM (S1 Table). These results argue against the clinical utility of the MDAPI to guide surgical decision making. Overall, our data suggests that patients in the BCT group received effective local and locoregional treatment. Interestingly, the majority of ME patients (71%) also presented with a low-risk MDAPI. We tried to identify reasons for ME in this group and identified 20 patients with multicentric tumors and 57 patients with cT3/cT4 tumors before NAC (34 of them with ypT3/ypT4 after NAC). It might be interesting to evaluate the use of the MDAPI to identify patients with low risk of LRR to prevent local overtreatment. However, an extensive DCIS component or micro-calcifications as well as patients’ choice could also have contributed to this observation and these cases may not be amenable to less radical surgery.

The cumulative 5-year DM risk was about twice as high as the LRR risk (19% *vs*. 10%). Interestingly, MDAPI led to a more distinct stratification of risk groups for DM (low: 14%; intermediate: 34% and high-risk: 61%; p<0.001) than for LRR (low: 8%, intermediate: 19% and high-risk: 23%; p = 0.03). Only 25 of the 40 patients with local failure presented with isolated LRR most of them occurring in the low-risk group (n = 19), only 6 in the intermediate–risk and none in the high-risk group. Of note, 5 events within the intermediate-risk group occurred in ME patients and could not have been prevented by adapting surgical strategy. All of the 6 patients received complete axillary lymph node dissection. As the prevention of local failure is likely to benefit only those patients without prior or simultaneous DM this limits the clinical utility further, as none of the patients in the MDAPI high-risk group developed isolated LRR. Prognosis in these patients is dominated by distant recurrences.

A general problem in our study was the skewed distribution of patients between MDAPI risk-groups, with only 2.7% MDAPI high-risk patients. This is similar to the initial report from the MD Anderson Cancer Center, with 83% low-risk and only 3.6% high-risk patients (none with MDAPI 4).[5] However, this initial study only reported on BCT patients, which inevitably leads to a shift to lower scores. The two validation studies that also included patients with ME and BCT, demonstrated considerably larger and clinically more relevant high-risk groups (Huang et al.: 10%; Akay et al.: 13%).[6,8] In both studies higher ME rates were observed compared to our study (both 59% *vs*. 42%).[6,8] These differences might reflect changes in the use of neoadjuvant chemotherapy over time, which was initially mostly used for locally advanced PBC but has now been accepted as a standard for early breast cancer.[12,13] The studies of Huang et al. and Akay et al. included patients treated between 1974–2000 and 2001–2005, respectively, whereas patients in our study were treated between 2003–2011. The introduction of (neo)adjuvant trastuzumab for HER2+ patients in 2005/2006 might also have influenced MDAPI score distribution to some extent. Our study is an observational study. Our major aim was to describe the clinical utility of the MDAPI score in the investigated patient collective, reported findings should be validated in future, independent trials.

Given recent advance in molecular profiling, this information should be included into the MDAPI either by subgroup-analysis or by an extension of the MDAPI.

In conclusion, our data do not confirm a clinical utility of the MDAPI to guide local therapy in patients with PBC. This is mainly due to extremely low numbers of patients in the high-risk category as well as the competing risk of distant metastasis dominating prognosis in these patients.

## Supporting information

S1 Table 5-year LR (A), 5-year LRR (B), 5-year LRR without DM (C) and 5-year DM rates (D) stratified by MDAPI risk groups and surgical procedure.
(PDF)

S2 Table Results from univariate and multiple survival analyses on LRRw/oDM. Abbreviations: HR: Hazard ratio; CI: confidence interval.
Bold represents probability values under 0.05
(PDF)

S3 Table De-identified data set. De-identified data set that was used to reach the conclusions drawn in the manuscript
(XLS)
