# Supplementary material for: Prediction of local recurrence risk after neoadjuvant chemotherapy in patients with primary breast cancer: Clinical utility of the MD Anderson Prognostic Index
Source: PLoS One. 2019 Jan 31;14(1):e0211337. doi: 10.1371/journal.pone.0211337 (PMC6355200; doi:10.1371/journal.pone.0211337)
Supplement: S1 Table — 5-year LR (A), 5-year LRR (B), 5-year LRR without DM (C) and 5-year DM rates (D) stratified by MDAPI risk groups and surgical procedure. (PDF) [file pone.0211337.s001.pdf]

S1 Table 5-year LR (A), 5-year LRR (B), 5-year LRR without DM (C) and 5-year DM rates (D) stratified by MDAPI risk groups and surgical procedure

A

MDAPI	Number of patients n	%	LR n	5-J-LR %	95% CI		pvalue
**Overall**	450	100.0	24	0.06	0.04	0.09	
**Low-risk**	374	83.1	14	0.05	0.02	0.07	0.005
**Intermed.-risk**	64	14.2	9	0.16	0.06	0.25	
**High-risk**	12	2.7	1	0.11	0	0.3	
**BET**	260	100.0	11	0.05	0.02	0.08	
**Low-risk**	239	91.9	8	0.04	0.01	0.07	0.05
**Intermed.-risk**	18	6.9	3	0.17	0	0.32	
**High-risk**	3	1.2	0	0	0	0	
**ME**	190	100.0	13	0.08	0.04	0.13	
**Low-risk**	135	71.0	6	0.05	0.01	0.09	0.1
**Intermed.-risk**	46	24.2	6	0.16	0.03	0.27	
**High-risk**	9	4.8	1	0.15	0	0.39	

B

MDAPI	Number of patients n	%	LRR n	5-J-LRR %	95% CI		pvalue
**Overall**	450	100.0	40	0.1	0.07	0.13	
**Low-risk**	374	83.1	27	0.08	0.05	0.11	0.03
**Intermed.-risk**	64	14.2	11	0.19	0.08	0.28	
**High-risk**	12	2.7	2	0.23	0	0.46	
**BET**	260	100.0	21	0.09	0.05	0.13	
**Low-risk**	239	91.9	17	0.08	0.04	0.12	0.3
**Intermed.-risk**	18	6.9	4	0.19	0	0.36	
**High-risk**	3	1.2	0	0	0	0	
**ME**	190	100.0	19	0.12	0.06	0.17	
**Low-risk**	135	71.0	10	0.09	0.03	0.15	0.1
**Intermed.-risk**	46	24.2	7	0.18	0.05	0.3	
**High-risk**	9	4.8	2	0.27	0	0.53	

C

MDAPI	Number of patients n	%	LRR w/o DM n	5-J-LRR w/o DM %	95% CI		pvalue
**Overall**	450	100.0	25	0.07	0.04	0.09	
**Low-risk**	374	83.1	19	0.06	0.03	0.09	0.3
**Intermed.-risk**	64	14.2	6	0.11	0.02	0.2	
**High-risk**	12	2.7	0	0	0	0	
**BET**	260	100.0	15	0.07	0.03	0.11	
**Low-risk**	239	91.9	14	0.07	0.03	0.11	0.99
**Intermed.-risk**	18	6.9	1	0.06	0	0.17	
**High-risk**	3	1.2	0	0	0	0	
**ME**	190	100.0	10	0.06	0.02	0.1	
**Low-risk**	135	71.0	5	0.04	0	0.08	0.2
**Intermed.-risk**	46	24.2	5	0.13	0.02	0.24	
**High-risk**	9	4.8	0	0	0	0	

D

MDAPI	Number of patients	DM	5-J-DM	95% CI	pvalue

	n	%	n	%			
**Overall**	450	100.0	94	0.19	0.15	0.22	
**Low-risk**	374	83.1	61	0.14	0.11	0.18	<0.001
**Intermed.-risk**	64	14.2	26	0.34	0.22	0.44	
**High-risk**	12	2.7	7	0.61	0.21	0.81	
**BET**	260	100.0	38	0.13	0.8	0.17	
**Low-risk**	239	91.9	30	0.11	0.06	0.15	0.005
**Intermed.-risk**	18	6.9	6	0.23	0.04	0.39	
**High-risk**	3	1.2	2	0.72	0	0.95	
**ME**	190	100.0	56	0.27	0.19	0.33	
**Low-risk**	135	71.0	31	0.21	0.13	0.27	0.06
**Intermed.-risk**	46	24.2	20	0.38	0.23	0.51	
**High-risk**	9	4.8	5	0.58	0.09	0.8	
