# Supplementary material for: Prediction of local recurrence risk after neoadjuvant chemotherapy in patients with primary breast cancer: Clinical utility of the MD Anderson Prognostic Index
Source: PLoS One. 2019 Jan 31;14(1):e0211337. doi: 10.1371/journal.pone.0211337 (PMC6355200; doi:10.1371/journal.pone.0211337)
Supplement: S2 Table — Abbreviations: HR: Hazard ratio; CI: confidence interval. Bold represents probability values under 0.05 (PDF) [file pone.0211337.s002.pdf]

S2 Table Results from univariate and multiple survival analyses on LRRw/oDM

				Univariate				Multiple			
**Variable**	**Level**	**Patients**	**Events**	**HR**	**95% CI**		**Pvalue**	**HR**	**95% CI**		**Pvalue**
Multifocal	0	376	18	Ref.			0.06	Ref.			**0.008**
	1	74	7	2.31	0.96	5.53		3.41	1.37	8.49	
MDAPI	0	220	11	Ref.			0.70				
	1	154	8	0.97	0.39	2.41					
	2	64	6	1.91	0.71	5.18					
	3	9	0								
	4	3	0								

Abbreviations: HR: Hazard ratio; CI: confidence interval
Bold represents probability values under 0.05
